# Supplementary material for: Piper nigrum Extract: Dietary Supplement for Reducing Mammary Tumor Incidence and Chemotherapy-Induced Toxicity
Source: Foods. 2023 May 19;12(10):2053. doi: 10.3390/foods12102053 (PMC10216990; doi:10.3390/foods12102053)
Supplement: Supplementary file 1 [file foods-12-02053-s001.zip › foods-2377335-supplementary.pdf]

Table S1. The components identified in PFPE by GC-MS and bioactivity.

	RT	Name of the compound	Molecular formula	Molecular Weight	Peak area (%)	Biological activity
Terpene (27.12%)						
1	18.929	Cyclohexene, 4-ethenyl-4-methyl-3-(1-methylethenyl)-1-(1-methylethyl)-, (3R-trans)- (or beta-Elemene)	$C_{15}H_{24}$	204.35	0.21	• Anticancer activity on K562 leukemic cells via induce cell apoptosis [24].
2	19.953	Copaene	$C_{15}H_{24}$	204.36	2.27	• Anti-microbial effect on anaerobic microorganism (*Prevotella nigrescens*) [25].<br>• Increases the antioxidant capacity in human lymphocyte cultures [26].
3	20.336	(1R,2S,6S,7S,8S)-8-Isopropyl-1-methyl-3-methylenetricyclo[4.4.0.02,7]decane-rel- (or β-Copaene)	$C_{15}H_{24}$	204.35	0.21	• No report
4	20.379	1,5,9-Cyclododecatriene, 1,5,9-trimethyl-	$C_{15}H_{24}$	204.35	0.16	• No report
5	21.101	Caryophyllene	$C_{15}H_{24}$	204.36	12.52	• Suppression of atherosclerosis development [27]<br>• Antioxidant and lipid oxidative damage prevention [28].<br>• Enhancing insulin release [29].<br>• Anti-genotoxicity [30].<br>• Anticancer activity of tumors developed from orthotopically grafted colon cancer cells into nude mice [31].
6	21.336	1H-Cyclopenta[1,3]cyclopropa[1,2]benzene, octahydro-7-methyl-3-methylene-4-(1-methylethyl)-, (3aS (3a.alpha.,3b.beta.,4.beta.,7.alpha.,7aS*)]- (or beta-Cubebene)	$C_{15}H_{24}$	204.35	0.14	• Cytotoxicity on MDA-MB-231 [32].
7	21.951	1,4,7,-Cycloundecatriene, 1,5,9,9-tetramethyl-, Z,Z,Z-	$C_{15}H_{24}$	204.35	0.68	• No report
8	22.770	ß-Selinene	$C_{15}H_{24}$	204.35	0.33	• Selective cytotoxic activity on MDA-MB-231 breast tumor cells [33].<br>• Anti-plasmodial activity and anti-cancer [34].
9	22.989	2-Isopropenyl-4a,8-dimethyl-1,2,3,4,4a,5,6,8a-octahydronaphthalene (or (-)-alpha-Selinene)	$C_{15}H_{24}$	204.35	0.41	• No report
10	23.107	α-Muurolene	$C_{15}H_{24}$	204.35	0.47	• Larvicidal activity [35].
11	23.299	ß-Bisabolene	$C_{15}H_{24}$	204.35	2.99	• Anti-cancer in MCF-7, MDA-MB-231, SKBR3 and BT474, and 4T1 mammary tumors *in vivo* [36].

	RT	Name of the compound	Molecular formula	Molecular Weight	Peak area (%)	Biological activity
12	23.481	(3S,3aR,3bR,4S,7R,7aR)-4-Isopropyl-3,7-dimethyloctahydro-1H-cyclopenta[1,3]cyclopropa[1,2]benzen-3-ol (or Cubebanol)	$C_{15}H_{26}O$	222.37	1.07	• No report
13	23.663	delta-Cadinene	$C_{15}H_{24}$	204.35	1.06	• Anti-cancer *in vitro* and *in vivo*[37]., anti-malarial [38].<br>• Anti-bacterial activity [39].
14	25.086	(-)-5-Oxatricyclo[8.2.0.0(4,6)]dodecane,,12-trimethyl-9-methylene-, [1R-(1R*,4R*,6R*,10S*)]- (or beta-Caryophyllene oxide)	$C_{15}H_{24}O$	220.35	0.90	• Anti-cancer via PI3K/AKT/mTOR/S6K1 and STAT3 pathways [40].<br>• Antioxidant [41].<br>• Anti-microbial activity [42].<br>• Inhibit PI3K/AKT/mTOR/S6K1 and MAPK pathways in human prostate and breast cancer cells [43].
15	26.118	Isospathulenol	$C_{15}H_{24}O$	220.36	0.36	• Anti-bacterial activity [44].
16	26.300	10,10-Dimethyl-2,6-dimethylenebicyclo[7.2.0]undecan-5.beta.-ol	$C_{15}H_{24}$	204.35	0.28	• No report
17	26.416	Muurolol	$C_{15}H_{26}O$	222.37	0.32	• Anti-insect and Anti-microbial activities[45].
18	26.508	1-Naphthalenol, 1,2,3,4,4a,7,8,8a-octahydro-1,6-dimethyl-4-(1-methylethyl)-, [1S-(1.alpha.,4.alpha.,4a.beta.,8a.beta.)]- (or Cadin-4-en-10-ol, Cadinol)	$C_{15}H_{26}O$	222.37	1.66	• Anti-cancer in breast MCF-7 cells [46].<br>• Antioxidant and cytotoxic activities in leukemic (HL-60, K562, and Jurkat) and solid tumor cells (MCF-7, HepG2, HT-1080, and Caco-2) [47].
19	53.209	gamma-Sitosterol (or clionasterol)	$C_{29}H_{50}O$	414.72	1.09	• Inhibitor of classical (CP) pathways of activation of the human complement system [48].
Amide (19.57%)						
20	32.461	2,4-Decadienamide, N-isobutyl-, (E,E)- (or Pellitorine)	$C_{14}H_{25}NO$	223.36	1.20	• Anti-inflammatory and anti-bacterial activity [49].
21	46.095	(2E,4E,10E)-N-Isobutylhexadeca-2,4,10-trienamide	$C_{20}H_{35}NO$	305.50	4.49	• No report
22	46.181	(2E,4E)-N-Isobutyloctadeca-2,4-dienamide (or Pipericine)	$C_{22}H_{41}NO$	335.58	4.54	• Hepatoprotective activity [50].
23	48.759	(2E,4E,14E)-N-Isobutylicosa-2,4,14-trienamide (or 2,4,14-Eicosatrienamide)	$C_{24}H_{43}NO$	361.61	8.32	• Cytoprotective activity in mouse L929 cells and hepatoprotective activity [50].
24	50.278	Octadeca-8,10-dien-12-ynoic acid, DMOX derivative	$C_{18}H_{28}O_2$	276.40	1.01	• No report

	RT	Name of the compound	Molecular formula	Molecular Weight	Peak area (%)	Biological activity
Alkaloid (52.82%)						
25	43.651	(2E,4E,6E)-7-(Benzo[d][1,3]dioxol-5-yl)-1-(piperidin-1-yl)hepta-2,4,6-trien-1-one (or Piperettine)	$C_{19}H_{21}NO_3$	311.40	2.22	• Anti-cholinesterase and Antioxidant effects [51].<br>• Trypanocidal effects against epimastigotes and amastigotes of *Trypanosoma cruzi* [52].
26	44.058	(E)-5-(Benzo[d][1,3]dioxol-5-yl)-1-(piperidin-1-yl)pent-2-en-1-one (or Piperanine)	$C_{17}H_{21}NO_3$	287.35	8.48	• Hepatoprotective activity [50].
27	44.405	Piperlonguminine	$C_{16}H_{19}NO_3$	273.33	0.53	• Anti-proliferation in lung cancer cells [53].<br>• Anti-cancer in breast cancer [54].<br>• Inhibits Pancreatic ductal adenocarcinoma (PDAC) cell proliferation *in vitro* and *in vivo* [55].
28	45.737	Piperine	$C_{17}H_{19}NO_3$	285.34	19.59	• Anticancer activity in DEN-induced HCC rats [56].<br>• Anti-hyperlipidaemic activity [57].<br>• Antitumor effect on cervical cancer cells [58].
29	45.871	Crinan, 1,2-didehydro-3-methoxy-, (3.alpha.)-	$C_{17}H_{19}NO_3$	301.34	1.06	• No report
30	46.010	N-Isobutyl-11-(3,4-methylenedioxyphenyl)-2E,4E,10E-undecatrienoic amide (or Pipercide)	$C_{22}H_{29}NO_3$	355.50	0.33	• Insecticidal activity in *Aedes aegypti* [59].
31	47.048	1-(Piperidin-1-yl)octadecan-1-one	$C_{23}H_{45}NO$	351.60	0.18	• No report
32	48.053	(E)-7-(Benzo[d][1,3]dioxol-5-yl)-1-(piperidin-1-yl)hept-6-en-1-one (or Piperolein A)	$C_{19}H_{25}NO_3$	315.40	4.94	• No report
33	48.657	(2E,6E)-7-(Benzo[d][1,3]dioxol-5-yl)-1-(piperidin-1-yl)hepta-2,6-dien-1-one (or Pipersintenamide)	$C_{19}H_{23}NO_3$	313.39	4.12	• Cytotoxic effect on CCRF-CEM, HL-60, PC-3, and HA22T cell lines [60].
34	49.369	(2E,4E)-1-(Piperidin-1-yl)octadeca-2,4-dien-1-one (or (2E,4E)-; Piperidine)	$C_{23}H_{41}NO$	347.58	0.16	• No report
35	49.909	Retrofractamide-A	$C_{20}H_{25}NO_3$	327.42	0.63	• Larvicidal activity against *Culex pipiens* pallens, Aedes aegypti and *A. togoi* [61].<br>• Hepatoprotective activity [50].<br>• Increased mRNA levels of adiponectin, peroxisome proliferator-activated receptor gamma 2 (PPARgamma 2), glucose transporter 4 (GLUT4), and insulin receptor substrate 1 (IRS-1) [62].

	RT	Name of the compound	Molecular formula	Molecular Weight	Peak area (%)	Biological activity
36	50.909	(E)-9-(Benzo[d][1,3]dioxol-5-yl)-1-(piperidin-1-yl)non-8-en-1-one (or Piperolein B)	$C_{21}H_{29}NO_3$	343.47	8.22	• Inhibitor of acyl CoA: diacylglycerol acyltransferase [63].<br>• Anti-fouling against cyprids of the barnacle *Balanus amphitrite* [64].<br>• Insecticidal activity [65].<br>• Activation of cation channel TRPV1 and TRPA1 [66].
37	51.840	(2E,4E,14E)-1-(Piperidin-1-yl)icosa-2,4,14-trien-1-one	$C_{25}H_{43}NO$	373.62	0.72	• No report
38	53.075	(2E,4E,8E)-9-(Benzo[d][1,3]dioxol-5-yl)-1-(piperidin-1-yl)nona-2,4,8-trien-1-one (or Dehydropipernonaline)	$C_{21}H_{25}NO_3$	339.40	0.38	• Anti-inflammatory [67].
39	55.226	(2E,4E,12E)-13-(Benzo[d][1,3]dioxol-5-yl)-N-isobutyltrideca-2,4,12-trienamide (or Guineensine)	$C_{24}H_{33}NO_3$	383.53	1.10	• Inhibitor of endocannabinoid uptake in BALB/c mice [68].<br>• Anti-inflammatory [69].
Lignan (0.49%)						
40	50.572	(3R,4R)-3-(Benzo[d][1,3]dioxol-5-ylmethyl)-4-(3,4-dimethoxybenzyl)dihydrofuran-2(3H)-one (or Kusunokinin)	$C_{21}H_{22}O_6$	370.40	0.49	• Anticancer activities both *in vitro* and *in vivo* [22,54,70-72].<br>• Insecticidal activity against *Virola sebifera* and fungicidal activity against *Leucoagaricus gongylophorus* [73].

Table S2. Classification of tumor histopathologic in the NMU-induced mammary tumoriogenesis rats of cancer treatment study.

Groups	Percentage of histological types	Histopathological of breast cancer tissue (H&E staining)	Explainations
Control	80%	Ductal carcinoma in situ, Grade III	Quickly grow, much variation of size and shape of nuclei, much difference from healthy breast cells, malignant cells completely fill the ducts [74].
	20%	Ductal carcinoma in situ, Grade I-III	Without invasive cells [74].
Vehicle	16.67%	Invasive ductal carcinoma Stage I, Grade I	With invading of cancer cells to surrounding fibrous or fatty breast tissues, low mitotic index (5-10 mitoses), normal tubular shape (>75%) and uniform nuclei with minimal nuclear variation in size and shape [74].
	66.66%	Ductal carcinoma in situ, Grade III	Quickly grow, much variation of size and shape of nuclei, much difference from healthy breast cells, malignant cells completely fill the ducts [74].
	16.67%	Ductal carcinoma in situ, Grade II	More variation of size and shape of nuclei, much microcalcifications [74].
Doxorubicin	16.67%	Ductal carcinoma in situ, Grade III	Quickly grow, much variation of size and shape of nuclei, much difference from healthy breast cells, malignant cells completely fill the ducts [74].
	16.67%	Ductal carcinoma in situ, Grade I	Growing at slow rate and much like normal breast cells [74].
	66.66%	No tumor	
PFPE100	100%	Ductal carcinoma in situ, Grade III	Quickly grow, much variation of size and shape of nuclei, much difference from healthy breast cells, malignant cells completely fill the ducts [74].
PFPE200	16.67%	Ductal carcinoma in situ, Grade I	Growing at slow rate and much like normal breast cells [74].
	50%	Ductal carcinoma in situ, Grade III	Quickly grow, much variation of size and shape of nuclei, much difference from healthy breast cells, malignant cells completely fill the ducts [74].
	33.33%	Intraductal papilloma, Grade 0	Benign or non-cancerous cells, fibrovascular core covered with epithelial and myoepithelial cells [74].
Dox+PFPE100	16.67%	Ductal carcinoma in situ, Grade I	Growing at slow rate and much like normal breast cells [74].
	33.33%	Ductal carcinoma in situ, Grade III	Quickly grow, much variation of size and shape of nuclei, much difference from healthy breast cells, malignant cells completely fill the ducts [74].
	50%	No tumor	
Dox+PFPE200	16.67%	Focal ductal hyperplasia, Grade I	Benign or non-cancerous cells originate at the milk ducts (Edge and Compton, 2010)
	16.67%	Ductal carcinoma in situ, Grade I	Growing at slow rate and much like normal breast cells [74].
	16.67%	Ductal carcinoma in situ, Grade III	Quickly grow, much variation of size and shape of nuclei, much difference from

Groups	Percentage of histological types	Histopathological of breast cancer tissue (H&E staining)	Explainations
			healthy breast cells, malignant cells completely fill the ducts [74].
	50%	No tumor	
Dox+PFPE300	16.67%	Ductal carcinoma in situ, Grade I	Growing at slow rate and much like normal breast cells [74].
	33.33%	Ductal carcinoma in situ, Grade II	More variation of size and shape of nuclei, much microcalcifications [74].
	50%	No tumor	
Dox+PFPE100+Tumeric25	100%	Ductal carcinoma in situ, Grade III	Quickly grow, much variation of size and shape of nuclei, much difference from healthy breast cells, malignant cells completely fill the ducts [74].
